# Silver Nanoparticles Produced by Laser Ablation and Re-Irradiation Are Effective Preventing Peri-Implantitis Multispecies Biofilm Formation

**DOI:** 10.3390/ijms231912027

**Published:** 2022-10-10

**Authors:** Ramón Pérez-Tanoira, Mónica Fernández-Arias, Carmen Potel, Raquel Carballo-Fernández, Sonia Pérez-Castro, Mohamed Boutinguiza, Miguel Górgolas, Fernando Lusquiños, Juan Pou

**Affiliations:** 1 Departamento de Microbiología, Hospital do Meixoeiro, Complejo Hospitalario Universitario de Vigo, 36312 Vigo, Spain; 2 Departamento de Microbiología, Hospital Universitario Príncipe de Asturias, 28805 Madrid, Spain; 3 Departamento de Biomedicina y Biotecnología, Universidad de Alcalá, 28801 Alcalá de Henares, Spain; 4 Departamento de Física Aplicada, Universidad de Vigo, 36310 Vigo, Spain; 5 Galicia Sur Health Research Institute (IIS Galicia Sur), SERGAS-UVIGO, 36213 Vigo, Spain; 6 Servicio de Enfermedades Infecciosas, IIS-Fundación Jiménez Díaz Hospital, 28040 Madrid, Spain

**Keywords:** nanoparticle synthesis, laser ablation, laser re-irradiation, particle size reduction, bacterial adhesion, biofilm formation

## Abstract

Implant-associated infection due to biofilm formation is a growing problem. Given that silver nanoparticles (Ag-NPs) have shown antibacterial effects, our goal is to study their effect against multispecies biofilm involved in the development of peri-implantitis. To this purpose, Ag-NPs were synthesized by laser ablation in de-ionized water using two different lasers, leading to the production of colloidal suspensions. Subsequently, part of each suspension was subjected to irradiation one and three times with the same laser source with which it was obtained. Ag-NPs were immobilized on the surface of titanium discs and the resultant materials were compared with unmodified titanium coupons. Nanoparticles were physico-chemically analysed to determine their shape, crystallinity, chemical composition, and mean diameter. The materials were incubated for 90 min or 48 h, to evaluate bacterial adhesion or biofilm formation respectively with *Staphylococcus aureus* or oral mixed bacterial flora composed of *Streptococcus oralis*, *Actinomyces naeslundii*, *Veionella dispar*, and *Porphyromonas gingivalis*. Ag-NPs help prevent the formation of biofilms both by *S. aureus* and by mixed oral bacterial flora. Nanoparticles re-irradiated three times showed the biggest antimicrobial effects. Modifying dental implants in this way could prevent the development of peri-implantitis.

## 1. Introduction

Infections caused by bacteria are behind a large number of complications in medical practice [1]. Patients with different pathologies are affected by infections due to bacterial contamination in medical devices [2]. In critical patients, a severe infection can cause death, but even in milder cases the harm to the patient is very high [3]. From an economic point of view, infections in medical devices cause a significant cost to the public health systems of the states [4]. In the US alone, spending on health care-associated infections is valued at more than $9.8 billion annually [5]. It is estimated that 80% of infections due to bacteria are due to the formation of a biofilm [6]. Biofilm formation leads to the development of long-lasting infections that are resistant to conventional antibiotic-based bactericidal treatments [7,8].

**Figure 1:**
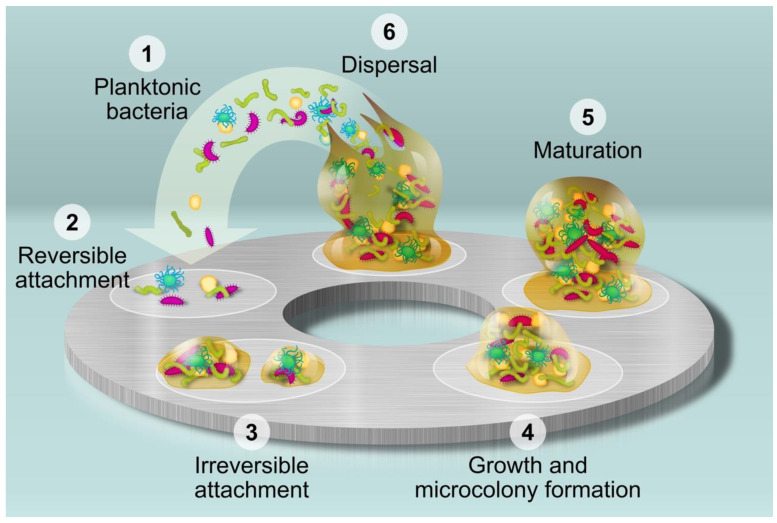
Main phases of the formation of biofilm on the surface of an implant.

The bacterial biofilm consists of a set of bacteria that adhere to a surface, develop, and form complex colonies that are held together by the extracellular matrix secreted by them [9]. Bacteria can form a biofilm on a multitude of different types of surfaces such as the surface of objects immersed in water, on the ground, on the surface of living tissues such as the lungs, ears, tooth enamel, or on the surface of medical devices, such as intraocular lenses, prosthetic joints, endotracheal tubes, intrauterine devices, peritoneal dialysis catheters, pacemakers, dental implants, etc. [9,10] Although every species of bacteria form a unique type of community, the main steps of biofilm formation appear to be maintained [11,12]. As depicted in Figure 1, the formation of biofilm on the surface of an implant follows several phases: adhesion to the surface (with reversible and irreversible attachment), proliferation (growth and microcolony formation), maturation, and dispersal [13].

When planktonic bacteria form aggregates or biofilms, they physically join and produce an extracellular matrix that is composed of various extracellular polymeric substances (EPS), such as proteins, polysaccharides, and DNA. This extracellular matrix keeps the bacteria together and protect them against antibiotics, making it really difficult to eliminate biofilm from an infected implanted medical device [14].

One of the most common inflammatory processes due to biofilm formation on a medical device is peri-implantitis. Osseo-integrated dental implants may be threatened by peri-implantitis, which is induced by the formation of bacterial biofilm in the oral cavity, being the most frequent cause of implant failure over time despite the effective prophylactic measures adopted [15,16,17]. Plaque accumulation produces progressive inflammatory destruction of the crest of the alveolar bone supporting the implant [18]. 

Peri-implantitis is a complex process caused by polymicrobial colonies that develop forming a biofilm on the surface of dental implants [18]. Prior to biofilm formation, early colonizing strains such as *Streptococcus* and *Staphylococcus* species adhere to the implant [19]. The adhesion mechanisms brought into play by bacteria such as *Staphylococcus aureus* promote their attachment to the implant surface. The development of peri-implantitis involves a change in the microflora from a predominantly gram-positive to a gram-negative microorganism [20,21].

One of the possible strategies to follow to avoid infections caused by biofilms is to prevent their formation. Taking into account the different stages in biofilm development shown in Figure 1, perhaps the best way to avoid biofilm formation is to prevent bacteria from adhering to the surface of the medical device [22,23].

The bactericidal properties of silver have been known for a long time. Metallic silver and silver ions have been used to prevent infection of chronic wounds or burns [24]. Coatings based on thin layers of silver have been tested and applied in different types of medical devices such as heart valves or catheters [25,26]. However, its use at the nanometric scale brings additional advantages because as the size of the material approaches the nanometric scale, its specific surface increases and new physical and chemical properties arise that are different from those of the massive material, which may be crucial in certain applications [27]. The use of Ag nanoparticles instead of massive silver means enhancing its properties, thanks to the increased release of Ag ions and greater contact with the surrounding medium, in addition to facilitating its penetration through the cell walls to attack pathogenic microorganisms from the inside, being able to locally destroy bacteria without affecting the healthy surrounding tissues [28,29,30]. Silver nanoparticles exhibit very promising results due to their excellent antibacterial, antifungal, and anti-inflammatory properties [31,32,33,34,35].

Although the exact mechanism responsible for the bactericidal effect of NPs is still poorly understood, the mode of action of NPs is direct contact with the bacterial cell wall. This makes most of the mechanisms of resistance to antibiotics deployed by bacteria irrelevant against NPs. That is why NPs arouse an enormous interest as an agent to combat dangerous antibiotic-resistant bacteria [36].

One important aspect that should be taken into account when using NPs against bacteria is that the antibacterial activity of NPs depends on particle size and shape [37,38,39].

For the production of silver nanoparticles, there are several different techniques available, based on chemical, physical, biological, and hybrid methods [40,41,42,43,44,45,46,47,48,49]. Obviously, each method has its own advantages and drawbacks. Selection of the individual method of production of silver nanoparticles (Ag-NPs) depends on the particular application. Several of these methods use precursor substances, solvents, or involve chemical reactions that can result in contamination of the nanoparticles. This fact is of special relevance when it comes to using nanoparticles as an alternative to antibiotics in a human being, given that these contaminating agents could be toxic, not only to bacteria, but also to other healthy cells that should not be harmed [50].

A method that allows obtaining pure nanoparticles without the need for the use of additional reagents is laser ablation in liquids (LAL) [51,52,53], also known as laser ablation synthesis in solution (LASiS) [54,55] or Pulsed Laser Ablation and excitation of nanoparticles in Liquids (PLAL) [56,57]. This method also allows the size and shape of the nanoparticles to be controlled by fine-tuning the processing parameters [58]. These characteristics make this technique a great alternative for the production of nanoparticles to combat infections in humans [59]. Laser ablation in liquids has been used quite frequently for the synthesis of silver nanoparticles by different researchers [60,61,62,63,64,65]. Variations of the original technique allowed improvements in the size of the nanoparticles or in the productivity of the method [66,67,68].

The use of silver nanoparticles obtained by laser ablation in liquid as a bactericidal agent has already been demonstrated by several researchers in different types of bacteria, including both gram-positive bacteria such as *Staphylococcus aureus* [69,70,71,72,73,74,75] or *Bacillus subtilis* [39,70,73,76] and gram-negative bacteria such as *Escherichia coli* [38,70,71,73,74,76,77,78] or *Pseudomonas aeruginosa* [69,70,71,73,74]. However, to the best of the authors’ knowledge, no works have been devoted to study the effect of silver nanoparticles obtained by laser ablation in liquids to prevent the development of multispecies biofilm.

The objective of this work is to study the effectivity of silver nanoparticles synthesized by laser ablation and re-irradiation in liquid against formation of multispecies biofilm involved in the development of oral implants peri-implantitis. This multispecies biofilm model includes the highly relevant oral bacterial species *Streptococcus oralis*, *Actinomyces naeslundii*, *Veillonella dispar,* and *Porphyromonas gingivalis*. In order to compare the effectiveness of the Ag-NPs against biofilm formation, single-bacteria (*Staphylococcus Aureus*) biofilm and the multispecies biofilm were tested.

## 2. Results

### 2.1. Physico-Chemical Characterization of the Nanoparticles

**Figure 2:**
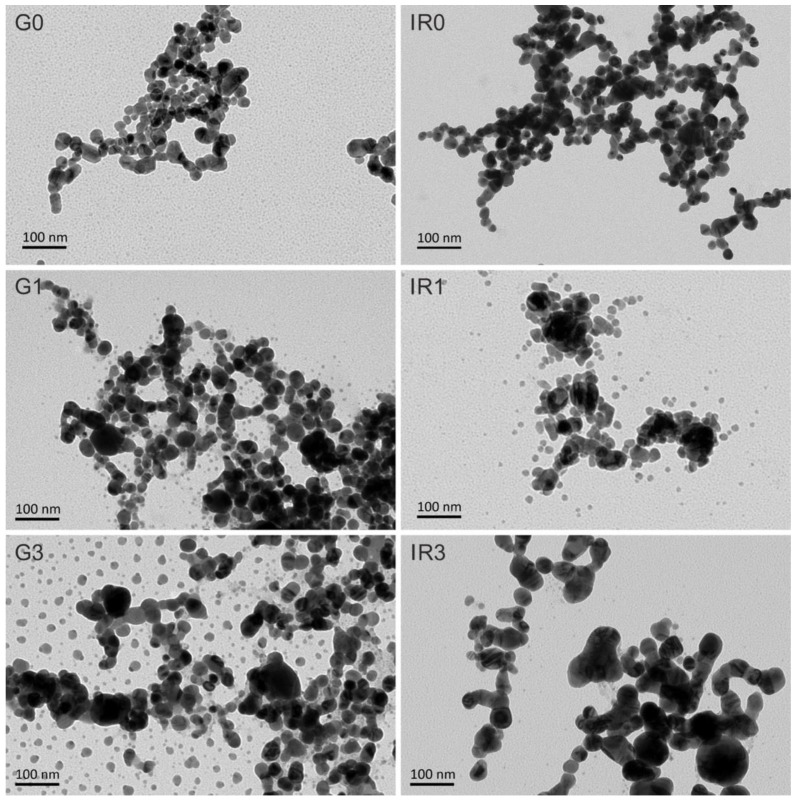
TEM micrographs of Ag nanoparticles obtained in water: (**G0**) by laser ablation with Green-nanosecond laser, (**IR0**) by laser ablation with IR-picosecond laser, (**G1**) after one re-irradiation with Green-nanosecond laser, (**IR1**) after one re-irradiation with IR-picosecond laser, (**G3**) after three re-irradiations with Green-nanosecond laser, and (**IR3**) after three re-irradiations with IR-picosecond laser.

Figure 2 shows transmission electron microscopy (TEM) images of Ag nanoparticles synthesized with both lasers: the Green-Nanosecond laser (Figure 2(G0,G1,G3)) and the IR-Picosecond one (Figure 2(IR0,IR1,IR3)). Images were taken on as-ablated nanoparticles (Figure 2(G0,IR0)) and after one (Figure 2(G1,IR1)) and three re-irradiations (Figure 2(G3,IR3)) made with the same laser.

As can be seen from the images shown in Figure 2, the specific laser processing conditions used and the number of irradiations directly influence the shape and size of the nanoparticles. A tendency towards agglomeration of the nanoparticles, forming chains, can be clearly observed. Only the effect of re-irradiation (Figure 2(G1,IR1,G3,IR3)) allows these chains to be broken, resulting in the formation of smaller nanoparticles. 

**Figure 3:**
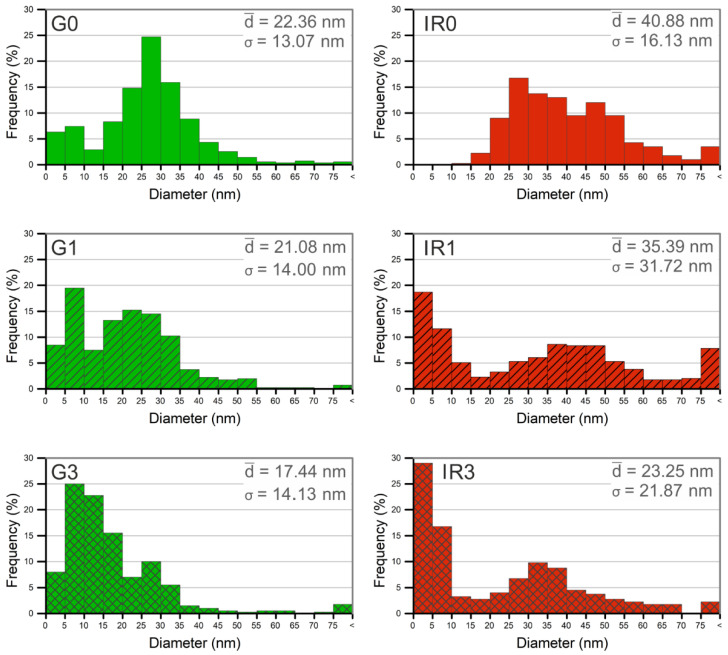
Size distribution of Ag nanoparticles obtained in water: (**G0**) by laser ablation with Green-nanosecond laser, (**IR0**) by laser ablation with IR-picosecond laser, (**G1**) after one re-irradiation with Green-nanosecond laser, (**IR1**) after one re-irradiation with IR-picosecond laser, (**G3**) after three re-irradiations with Green-nanosecond laser, and (**IR3**) after three re-irradiations with IR-picosecond laser.

In order to perform a quantitative evaluation of the diameters of the nanoparticles synthesized under different conditions, the size of more than 400 nanoparticles was measured. For this, transmission electron microscopy images of each type of sample were used. The histograms in Figure 3 show the resulting diameter distributions. From the analysis of these histograms, it is clearly deduced that the average diameter of the nanoparticles is smaller when using the green laser than the IR one (a result that is repeated regardless of the number of re-irradiations). Likewise, the re-irradiation of the recently ablated nanoparticles produces a reduction in their average diameter. The diameter reduction becomes more marked as the number of re-irradiations increases, and this trend is repeated regardless of the type of laser used.

The fact that, in all the cases studied, the mean size of the nanoparticles obtained using the green-nanosecond laser are smaller than in the case of the IR-picosecond laser can be explained by the greater absorption cross-section that silver presents at shorter wavelengths [58]. However, in addition, the greater fluence of the ablation process carried out with the green laser influences the reduction of the average diameter of the NPs [79]. This is especially relevant when the incident radiation affects a small controlled area as is the case here.

The re-irradiation of nanoparticles, regardless of the type of laser used, produces a large number of small-diameter particles and, at the same time, generates nanoparticles with a broader size range. Note that all re-irradiated samples (Figure 3(G1,IR1,G3,IR3)) show a bimodal size distribution. The metallic nature of the precursor material (silver) causes that the re-irradiated material tends to coalesce, which explains this bimodal behaviour of the diameter distributions. The vast majority of nanoparticles are fractionated by the action of the laser beam, converting them into smaller particles. However, at the same time, the energy absorbed by these nanoparticles causes their temperature to increase above the melting point. When several of these liquid nanoparticles come into contact with each other, they coalesce, giving rise to larger particles and the formation of chain-shaped nanostructures [80].

In addition to size, other characteristics such as surface charge are related to the bactericidal activity of nanoparticles [81]. Measuring the Z-Potential (ζp) is an easy, fast, and economic way to measure the surface charge changes. In this sense, the ζp of each sample was measured after being obtained (see Table 1).

As can be clearly visible in Table 1, all values are negative being the absolutes higher in case of particles obtained by laser ablation than the re-irradiated, decreasing with the number of re-irradiations which is related to an increased instability, being the colloidal solutions with ζp values 20 mV more stable than the ones with ζp below 10 mV [82].

**Figure 4:**
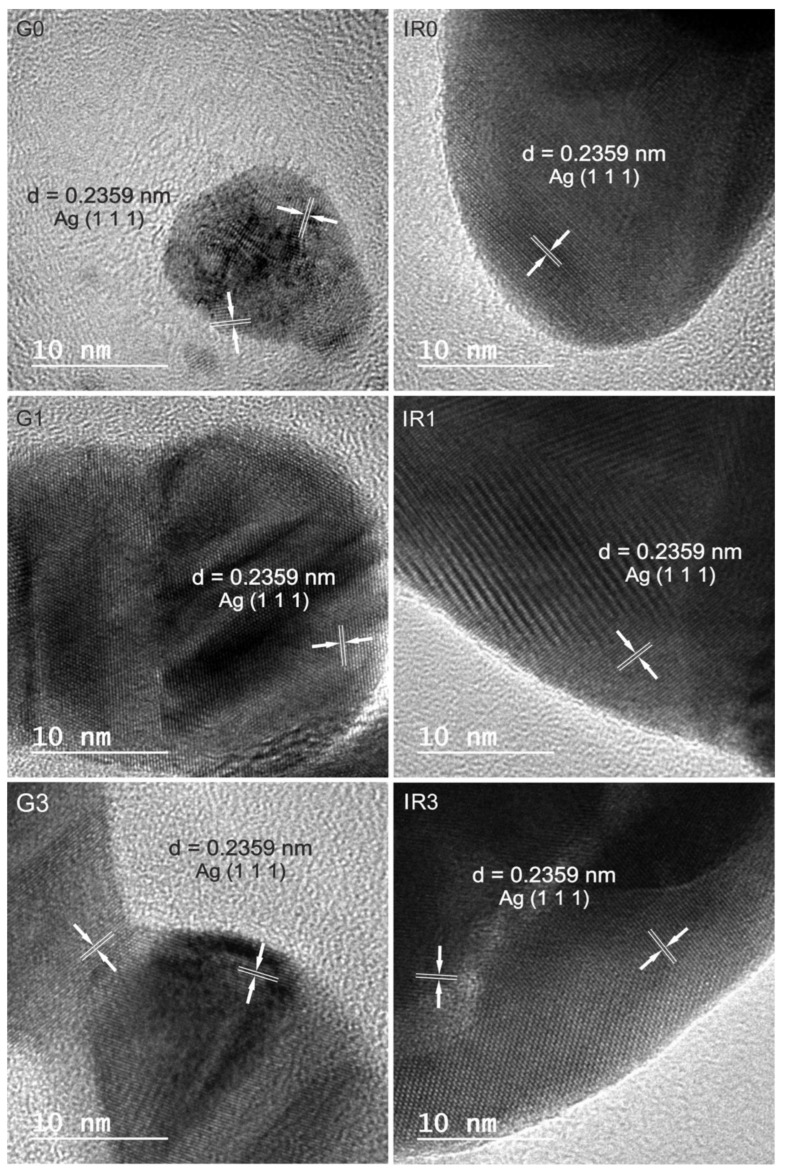
HRTEM images of Ag crystalline nanoparticles obtained in water (**G0**) by laser ablation with Green-nanosecond laser, (**IR0**) by laser ablation with IR-picosecond laser, (**G1**) after one re-irradiation with Green-nanosecond laser, (**IR1**) after one re-irradiation with IR-picosecond laser, (**G3**) after three re-irradiations with Green-nanosecond laser, and (**IR3**) after three re-irradiations with IR-picosecond laser.

Regarding the chemical composition of the nanoparticles obtained, the energy dispersive spectroscopy (EDS) analyses carried out on all the groups of nanoparticles obtained, both the as-ablated and the re-irradiated ones, gave the result that they are made up of pure silver. On the other hand, all nanoparticles are crystalline, even the smallest ones, as can be observed in Figure 4. This typical crystalline aspect is shown by all nanoparticles obtained, independently of the type of laser used and the number of re-irradiations. No sign of amorphization or formation of an oxide layer can be observed.

Regarding the chemical composition of the nanoparticles obtained with the different lasers and after different re-irradiations, the interplanar distances measured in the corresponding high-resolution transmission electron microscopy (HRTEM) images confirm that the predominant lattice spacing can be indexed to cubic Ag. 

**Figure 5:**
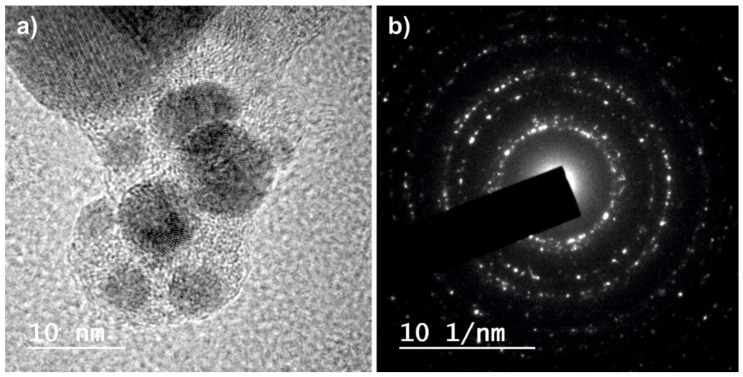
(**a**) HRTEM image and (**b**) SAED of Ag crystalline nanoparticles obtained in water after three re-irradiations with IR-picosecond laser. The diffraction peaks from A, B, C, D, are consistent with Ag, showing 0.235, 0.204, 0.144, 0.123, 0.118, 0.101 nm, and the corresponding (1 1 1), (2 0 0), (2 2 0), (3 1 1), (2 2 2), (4 0 0) family planes of silver.

To identify the crystalline phases present in each group of nanoparticles, the selected area electron diffraction (SAED) pattern was obtained in several groups of particles as shown in Figure 5.

Figure 5a shows an image obtained by HRTEM of a group of nanoparticles obtained by means of the IR-picosecond laser after three re-irradiations. The SAED performed on this group of particles is shown in Figure 5b. The concentric diffraction rings with bright spots are clearly seen in this pattern. This is the typical crystalline aspect shown by all nanoparticles obtained independently of the type of laser used and the number of re-irradiations.

The interplanar distances obtained by SAED are compared with those of metallic Ag in Table 2. The interplanar distances measured correspond to the families of planes of cubic Ag (JCPDS-ICDD ref. 00-004-0783).

**Figure 6:**
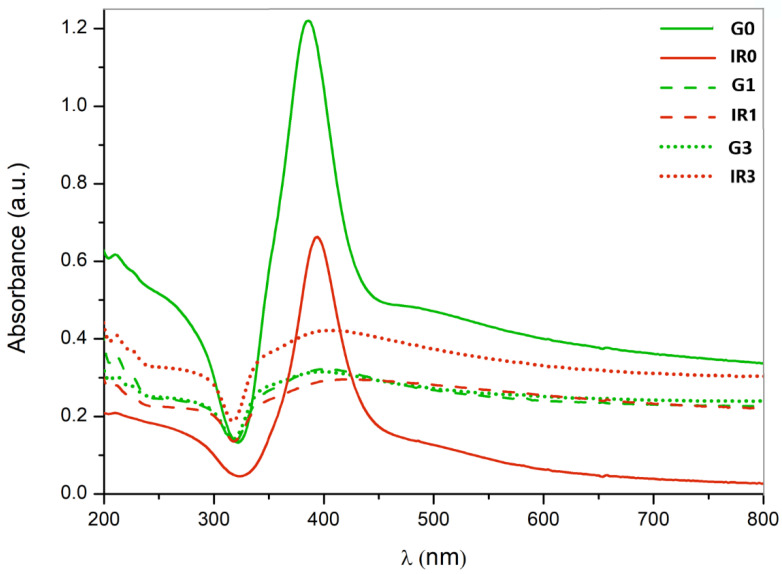
UV-VIS spectra of Ag nanoparticles obtained. Processing conditions correspond with those shown in Table 1.

Figure 6 shows the UV-VIS spectra of the six groups of silver nanoparticles synthesized by laser ablation in liquid according to what is specified in Table 3. All the groups of nanoparticles show a peak at approximately 400 nm. This peak is typical of the surface plasmon resonance (SPR) characteristic of Ag nanoparticles in colloidal solutions [83]. The SPR is a known effect for metallic nanoparticles, which is non-present both in the individual atoms and in the metallic bulk. This peak is correlated with both the form of the nanoparticles and their size, as well as to their surrounding medium. Thus, the presence of a single surface plasmon peak implies that nanoparticles are spherical [83,84]. The pronounced peak observed for the G0 and IR0 groups of nanoparticles (directly obtained after laser ablation) corroborates their sphericity observed by TEM (see Figure 2(G0,IR0). Meanwhile, the broadening in the UV-VIS spectra of the groups of re-irradiated nanoparticles (G1, G3, IR1, and IR3) corresponds to a broad size distribution [85] that is in agreement with the TEM observations (see Figure 2(G1,IR1,G3,IR3).

**Figure 7:**
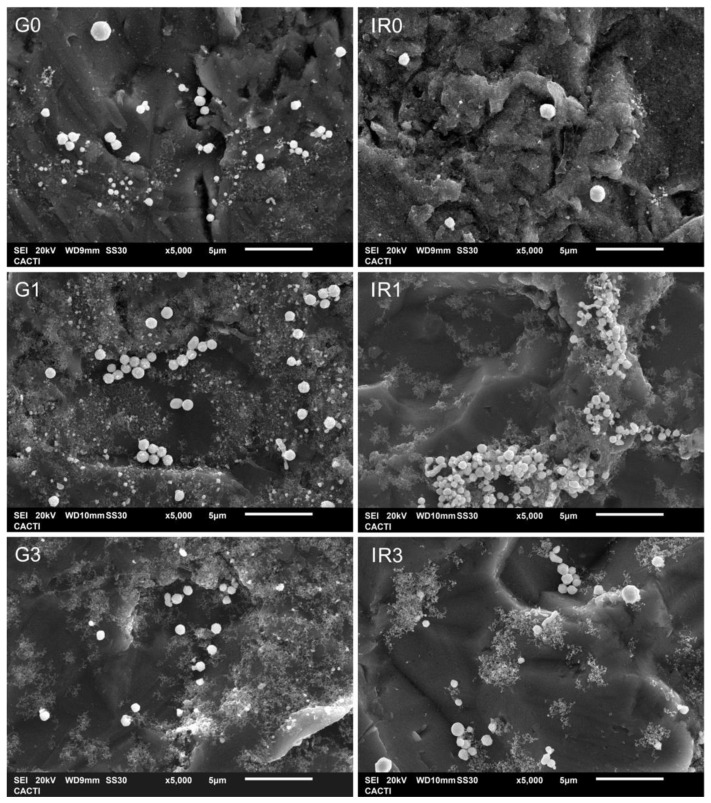
FESEM micrographs of titanium discs with immobilized Ag nanoparticles obtained in water: (**G0**) by laser ablation with Green-nanosecond laser, (**IR0**) by laser ablation with IR-picosecond laser, (**G1**) after one re-irradiation with Green-nanosecond laser, (**IR1**) after one re-irradiation with IR-picosecond laser, (**G3**) after three re-irradiations with Green-nanosecond laser, and (**IR3**) after three re-irradiations with IR-picosecond laser.

Once the different groups of nanoparticles were adequately characterized, they were immobilized on the surface of grade 2 pure titanium discs. Figure 7 shows a detail (×5000 magnification) of the surface of the titanium discs with the different groups of nanoparticles as observed by means of the field emission scanning microscope (FESEM). 

These figures show the distribution of the silver nanoparticles on the irregular surface of the titanium discs. The spherical shape of the nanoparticles can be corroborated, and the presence of nanoparticles of different sizes, especially that of the groups of re-irradiated nanoparticles (see Figure 7(G1,IR1,G3,IR3)), confirming the quantitative data of the diameter histograms shown in Figure 3.

**Figure 8:**
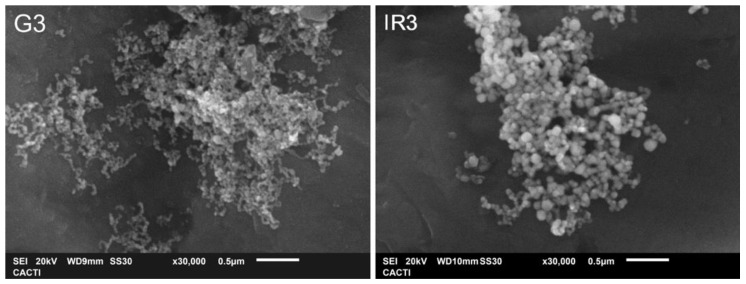
FESEM micrographs of titanium discs with immobilized Ag nanoparticles obtained in water by laser ablation after three re-irradiations with: (**G3**) Green-nanosecond laser, and (**IR3**) IR-picosecond laser.

In this sense, higher magnification (×30,000) was used to observe the smallest nanoparticles in detail. Figure 8 corroborates the nature of the cotton-like structures present in the samples.

### 2.2. Bacterial Adhesion Tests

**Figure 9:**
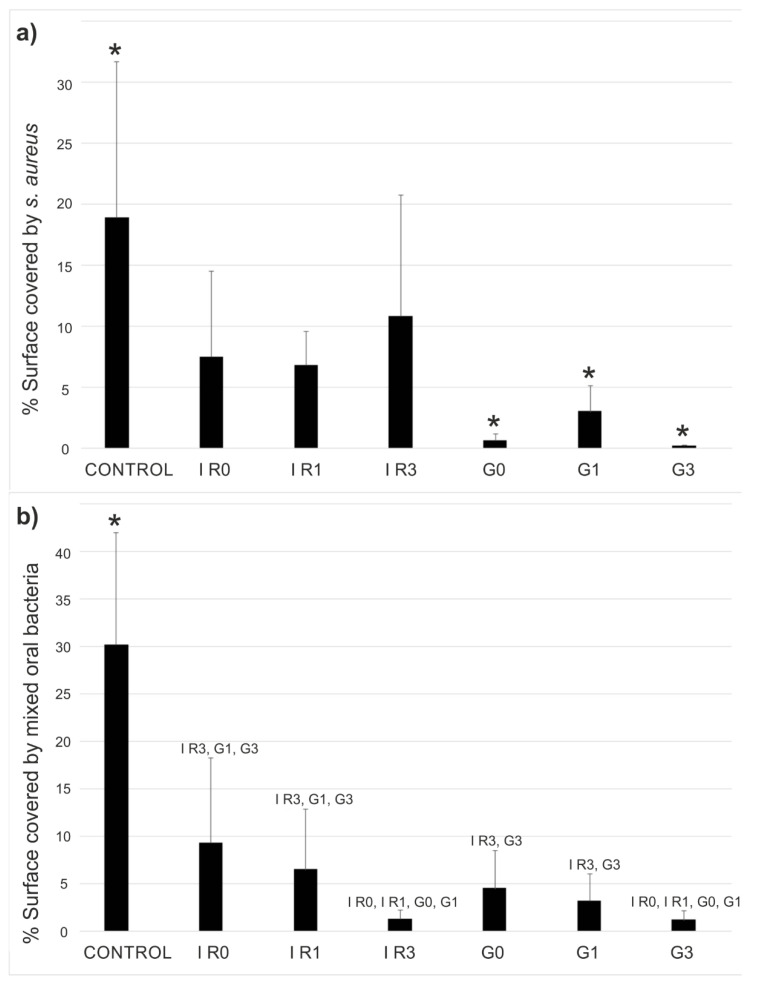
Bacterial adhesion. Results are given in mean percentage of titanium surface covered with (**a**) *S. aureus* or (**b**) mixed oral bacteria after 90 min of incubation. Control sample is the bare titanium disc without Ag-NPs. The other samples correspond to Ti discs with immobilized Ag-NPs obtained according to conditions of Table 3. The error bars represent the standard deviation (* *p* < 0.05 with respect all the other materials).

As illustrated in Figure 1, the first step on the formation of the biofilm on the surface of an implant is the adhesion of the planktonic bacteria on that surface. Therefore, we carried out experiments to study the ability of the silver nanoparticles to inhibit adhesion of *S. aureus* as well as the mixed oral bacteria on the titanium discs. Figure 9 shows the adhesion of both *S. aureus* and mixed oral bacteria to the titanium surface, in terms of percentage of surface covered by the bacteria. 

The adhesion results indicate that *S. aureus* and mixed oral bacteria showed lowered adherence to the titanium discs with immobilized Ag-NPs compared to untreated titanium surface (*p* ≤ 0.01). The incorporation of silver NPs resized by the green-nanosecond laser (Figure 9a sample G3) was more effective to inhibit the bacterial adhesion to the titanium surfaces, than using the IR-picosecond laser to re-irradiate the Ag-NPs (Figure 9a sample IR3). The Ag-NPs re-irradiated three times using the Green-nanosecond laser (samples G3) and immobilized on the Ti discs produced a higher anti-adherent effect (*p* ≤ 0.001) for all the comparisons except for *S. aureus* regarding G0 and for mixed oral bacteria regarding IR3 (*p* = 0.034 and *p* = 0.387 respectively). 

**Figure 10:**
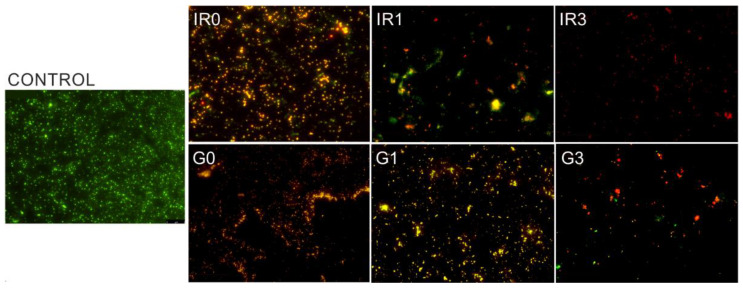
Bacterial adhesion. Images taken with the fluorescence microscope of mixed oral bacteria after 90 min of incubation. Control sample is the bare titanium disc without Ag-NPs. The other samples correspond to Ti discs with immobilized Ag-NPs obtained according to conditions of Table 3.

Figure 10 shows an example of the results of the tests performed using the live/dead bacterial viability kit. In this case, images obtained by the fluorescence microscope correspond to the adhesion tests of the mixed oral bacteria after 90 min of incubation.

This type of test allows assessing the viability of the bacteria as a function of the integrity of the cell membrane. Cells that present a damaged membrane that can be considered dead or dying, will be stained in red colour. While the cells that preserve an intact membrane appear stained in green colour [86]. Therefore, from a qualitative point of view, all six groups of silver nanoparticles immobilized on the titanium disc surfaces (samples G0, G1, G3, IR0, IR1, and IR3 of Figure 10) present an inhibitory activity as compared to the bare titanium disc (control sample of Figure 10). This is a remarkable result, taking into account that the mixed oral flora is formed by four different bacteria, two of them gram-positive (*Streptococcus oralis* and *Actinomyces naeslundii*) and the other two (*Veillonella dispar* and *Porphyromonas gingivalis*) are gram-negative. That means, even if the membranes are different, Ag-NPs are able to damage all tested bacteria, preventing their adhesion onto the titanium surface.

### 2.3. Biofilm

#### 2.3.1. Percentage of Surface Covered by Biofilm

**Figure 11:**
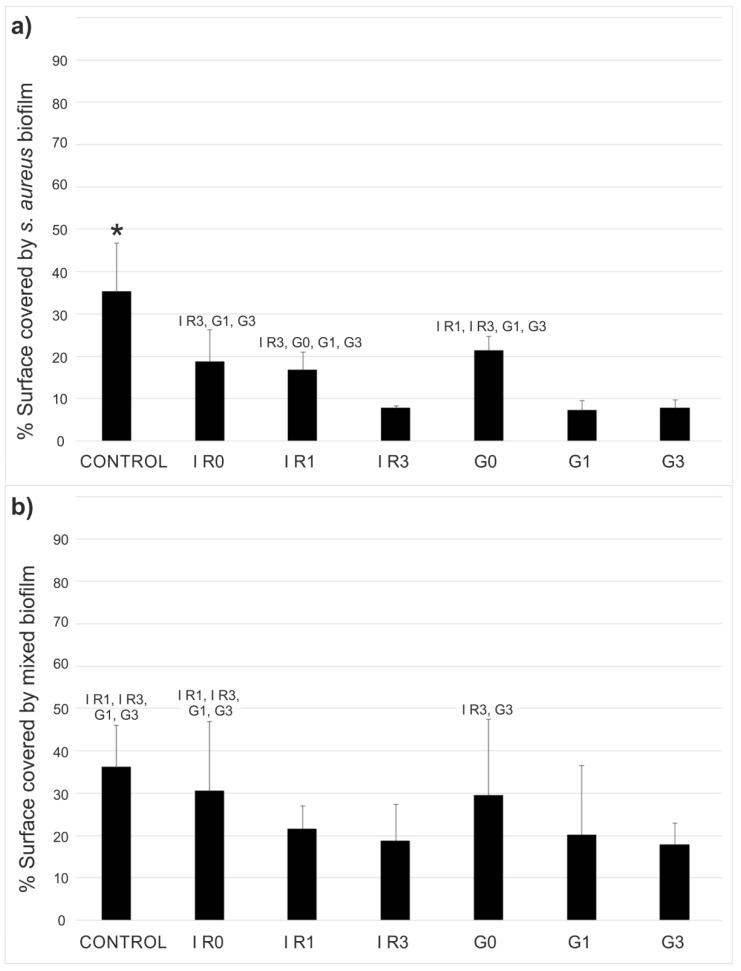
Biofilm inhibition test. Results are given in mean percentage of titanium surface covered with (**a**) *S. aureus* or (**b**) mixed oral bacteria after 48 h of incubation. Control sample is the bare titanium disc without Ag-NPs. The other samples correspond to Ti discs with immobilized Ag-NPs obtained according to conditions of Table 3. The error bars represent the standard deviation (* *p* < 0.05 with respect all the other materials).

After 48 h of incubation of titanium discs in presence of *S. aureus* or mixed oral bacteria, an anti-biofilm effect of silver was found due to the reduction of surface covered by both biofilms, as it is shown in Figure 11. This reduction was statistically significant (*p* ≤ 0.037) for *S. aureus* on all Ti discs with immobilized Ag-NPs (see Figure 11a). However, for mixed oral bacteria only the Ti discs with Ag-NPs re-irradiated one or three times (samples G1, G3, IR1, and IR3 of Figure 11b) shown statistically significant reduction of biofilm formation than the bare titanium (*p* ≤ 0.005). 

**Figure 12:**
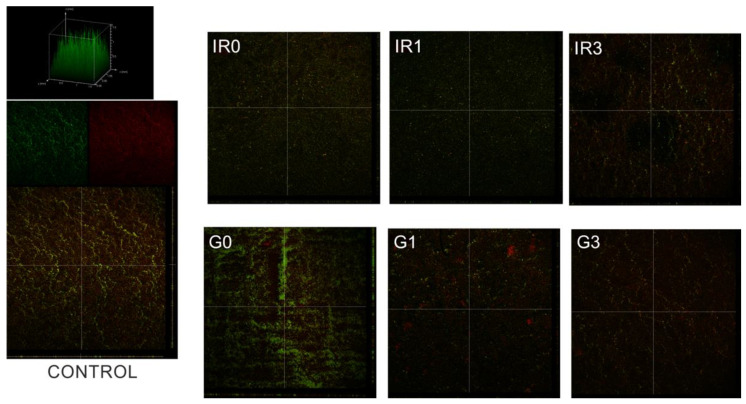
Biofilm inhibition test. Example of the fluorescence CLSM images for Ti surface covered by mixed oral bacteria biofilms for the different types of samples tested. Control sample is the bare titanium disc without Ag-NPs. The other samples correspond to Ti discs with immobilized Ag-NPs obtained according to conditions of Table 3.

Figure 12 shows an example of the fluorescence by confocal laser scanning microscope (CLSM) images obtained for mixed oral bacteria after 48 h of incubation, for the six different groups of samples tested, as well as the untreated titanium as control.

Samples IR3 and G3 showed a statistically significant reduction in the surface covered by both biofilms with respect to the other materials studied, except when compared with IR1 and G1 in the case of mixed oral bacteria biofilms.

#### 2.3.2. Thickness of Biofilm

**Figure 13:**
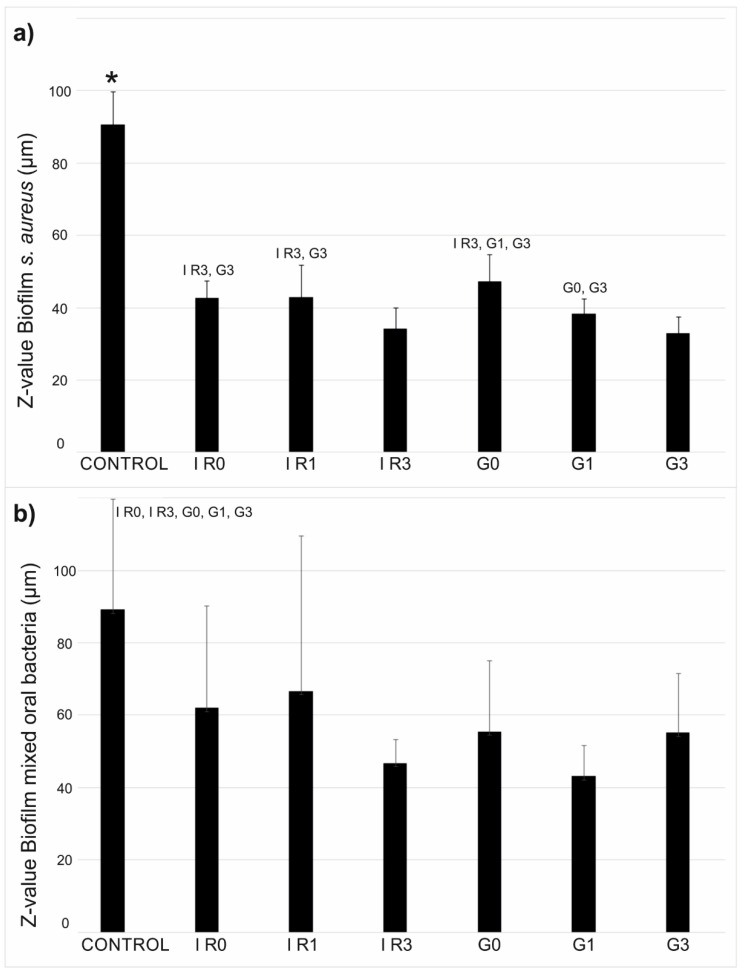
Mean thickness values of the biofilms of (**a**) *S. aureus* and (**b**) mixed oral bacteria. Control sample is the bare titanium disc without Ag-NPs. The other samples correspond to Ti discs with immobilized Ag-NPs obtained according to conditions of Table 3. The error bars represent the standard deviation (* *p* < 0.05 with respect all the other materials).

Figure 13 shows the mean thickness values of the biofilms formed by *S. aureus* (Figure 13a) and by the mixed oral bacteria (Figure 13b) on the bare titanium discs (control sample in Figure 13) and on the titanium discs with immobilized Ag-NPs obtained according to conditions of Table 3 (samples G0, G1, G3, IR0, IR1, and IR3 in Figure 13). Thickness of the biofilms is given in terms of z-value following the procedure described in Section 4 and developed by M. Müsken et al. [87].

The incorporation of silver on the surface of titanium discs diminished the mean values of the thickness of biofilms formed by *S. aureus* and mixed oral bacteria (*p* < 0.001 and *p* ≤ 0.02, respectively), with the exception of the mixed oral bacteria biofilm formed on IR1 (*p* = 0.174). The lowest z-value of *S. aureus* biofilm was obtained on samples IR3 and G3 (*p* ≤ 0.02), excepting the comparison of samples G1 and IR3 (*p* = 0.070). There were no significant differences for z-value of biofilms formed by mixed oral bacteria on all titanium discs with the different types of Ag-NPs.

## 3. Discussion

Since the control of dental plaque formation is an important prevention strategy of peri-implantitis, restorative materials with antibacterial properties are desirable to help to maintain the residual inactive microorganism. On the other hand, it is necessary to discover new antibiotics and develop alternative therapies that allow us to fight efficiently against the enormous challenge posed by the growing resistance of microbes to antibiotics [88]. In this study, the antibacterial effect of resized Ag-NPs obtained by re-irradiating with Green or Infrared laser an aqueous colloidal solution of silver a different number of times (one and three times) was compared with unmodified titanium discs. Finally, we evaluated which of the laser ablation conditions produced resized Ag-NPs with more important anti-adherent properties and antibiofilm effect.

Silver-modified surfaces showed a significant reduction in bacterial adherence and biofilm formation compared to untreated material. This indicates that the silver ions available on the titanium material surface lead to an improved antimicrobial effect. Titanium samples decorated with Ag-NPs obtained from green laser ablation showed a reduction of bacterial adherence compared to those obtained using the Infrared one. This was not the case for biofilm formation; irradiating the nanoparticles already formed with IR or green laser showed no advantages over each other. The most remarkable result is that the antimicrobial effect of silver was greater with Ag-NPs of a smaller size, which were obtained by subjecting their aqueous solution to an extra triple irradiation. This finding is in agreement with others previously reported in the literature [89,90]. The smaller particles have greater antibacterial activity due to their higher surface to volume ratio. The work by Duran et al. [91] showed that when the silver nanoparticles were larger than 10 nm, the predominant antimicrobial mechanism was the action of the silver ions released by the particles. However, when the diameter of the Ag-NPs was less than 10 nm, the antimicrobial activity was mainly due to the penetration of the nanoparticles through the bacterial membrane [91]. 

Another interesting result is that all the silver nanoparticles produced are highly stable and crystalline, regardless of the wavelength of the laser used for ablation and re-irradiation and the number of re-irradiations carried out. This fact of maintaining a crystalline microstructure, together with its small size, may be linked to its high bactericidal and antibiofilm effect [92].

As mentioned in Section 4, the method followed in this work to obtain the Ag-NPs is the same as the one already used by the authors [72]. In this previous work, Ag-NPs were obtained using two lasers: a green-nanosecond one, same as used here, and the second one was an infrared-nanosecond laser, that emits the laser radiation with pulses in the nanosecond range instead of the picosecond as is the case of the present work. Comparing the Ag-NPs produced in both works, we can affirm that nanoparticles show similar morphology, and the average size is close to identical when comparing groups of nanoparticles produced with the same laser wavelength and after the same number of re-irradiations. If we compare the average size of Ag-NPs produced with the IR-nanosecond laser [72] and the nanoparticles produced with the IR-picosecond one (this work), the results are close to identical among groups of nanoparticles produced after the same number of re-irradiations. The comparison of all groups from both works suggest that laser wavelength seems to play a more relevant role on the Ag-NPs average size than pulse duration. Nevertheless, more experiments need to be carried out to confirm this hypothesis. With regard to the bactericidal activity, in our previous work [72] studies were made with Ag-NPs in colloidal suspensions, whereas in the present work nanoparticles are immobilized on titanium discs. Results are not directly comparable, but in both cases the presence of silver nanoparticles has antibacterial activity, or prevents adhesion of bacteria and biofilm formation, when we compare against bacteria cultures carried out without the presence of Ag-NPs.

The mechanism of Ag-NPs’ activity on bacterial adhesion and biofilm formation is not fully understood yet. In general terms, there are four main mechanisms or routes of bactericidal action of silver nanoparticles: [92] their adhesion to the cell membrane, the penetration of nanoparticles into bacterial cells, the formation of reactive oxygen species, and the modulation of the pathway of cellular signal transduction by nanoparticles. Several authors agree that the nanoparticles adhere to the cell membrane, changing its morphology and its permeability. The silver ions react with electron donor groups (N, O, or S), which are present in bacteria in proteins, RNA, DNA, and chloride ions, inactivating bacterial enzymes by promoting the release of iron with the subsequent formation of hydroxyl radicals by an indirect mechanism probably mediated by reactive oxygen species [26,93]. Thus, on the one hand, it avoids the correct transport of substances through it, and on the other interacts with the sulphur and phosphorus that it contains resulting in metabolic failure, all leading to bacterial lysis [91,94]. Antimicrobial activity of silver takes place via the generation of reactive oxygen species, which induces permeability of the membrane and DNA transformation [95]. Small concentrations of silver ions are sufficient for microbicidal activity [96,97].

The oral multispecies biofilm model used in this study is a clear improvement on current methods, mimics the native situation, and is robust and highly reproducible [98]. CLSM analysis is a well-established quantitative system for estimating the bactericidal efficacy of antibacterial agents in biofilms [99]. Live/Dead BaclLight has been shown to be an effective stain to distinguish between active and dead bacteria and has enjoyed increasing popularity among researchers in various fields since it was released about 20 years ago [86]. The kit used has the particularity that distinguishes only between bacteria with damaged cytoplasmic membrane and those that keep it intact. Generally speaking, it seems correct to assume that membrane-intact bacteria are active cells [100,101]. However, if we take into account that there are antimicrobial substances that do not directly affect the cell membrane (for example, those that interfere with the synthesis of nucleic acids or proteins), which are considered bacteriostatic agents, we can affirm that this viability staining method is not universally valid for evaluating the antimicrobial activity of a substance [102,103]. On the other hand, intermediate states between viable and non-viable bacteria can occur, which needs to use more than one viability indicator for analysis [100,104]. For that reason, we investigated only the presence of bacteria and not the viability. 

We are aware that our research may have some limitations, such as the unknown effect of the extracellular compound coating the implant surface when it is in contact with the patient’s body fluids. Albumin or fibrinogen proteins, present in the extracellular fluid, can influence the results, because these proteins could boost or inhibit the adhesion of bacteria to the implant surface via specific ligand-receptor interactions [105,106]. Another limitation is that only bacterial collection strains were tested. These bacterial collections are cultured under controlled laboratory conditions, whereas strains obtained from prosthetic infections are somehow wild. Using these wild-type of bacterial strains would perhaps show greater variability, different pathogenic properties, and attach to the implant surface at different concentrations [107]. We need to study all technical parameters of ablation in as many as possible clinical strains to achieve more realistic results and to better learn bacterial behaviour. In order to resolve species-specific viability, more labour-intensive methods are required, such as propidium monoazide quantitative real-time polymerase chain reaction (PMA-) qRT-PCR and fluorescent in situ hybridization (FISH). Moreover, these techniques will allow to evaluate the spatial distribution of biofilms on the surface of implants, providing much more information on the interactions of bacteria and implant surfaces.

It is noteworthy to mention that the bactericidal assays carried out previously to analyse the inhibitory effects of the obtained Ag-NPs in colloidal suspension against *S. aureus* [72] are consistent in general terms with the present results. The obtained Ag nanoparticles show great antibacterial capacity, which is increased when the precursor colloidal solutions are re-irradiated three times. This is in agreement with the size reduction of precursor nanoparticles, indicating that nanoparticle size is crucial to increase their antibacterial effect [108].

## 4. Materials and Methods

### 4.1. Nanoparticle Production

**Figure 14:**
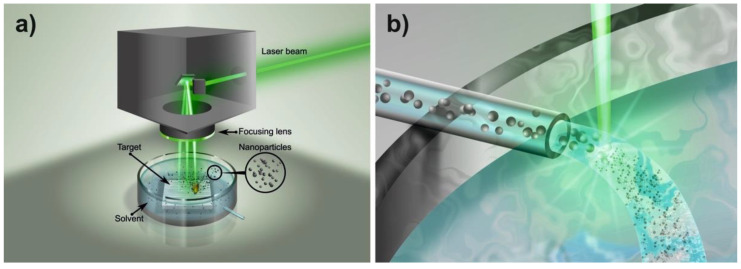
(**a**) Laser ablation in water and (**b**) re-irradiation method followed to produce Ag-NPs.

Silver NPs were synthesized by laser ablation and re-irradiation in water technique, (see Figure 14) following the method reported previously [72]. The procedure is briefly described below for more clarity. 

A silver foil with 99.99% of purity (Alfa Aesar, Thermo Fisher Scientific, Waltham, MA, USA), previously cleaned and sonicated, was immersed in 200 mL of de-ionized water, to be used as a target for the laser ablation process using two different diode-pumped Nd:YVO_4_ laser sources (Rofin-Sinar, Hamburg, Germany). Table 3 collects the sample designations. First, a visible laser was used (wavelength of 532 nm, which corresponds to the green band of the electromagnetic spectrum). This laser provided pulses of 0.26 mJ of energy and with a duration of 14 ns.

The second laser source used provided pulses of 0.03 mJ of energy, with a duration of 800 ps and a wavelength of 1064 nm, which corresponds to the infrared (IR) range of the electromagnetic spectrum. In both cases, the laser beam was focused on the surface of the silver target, obtaining a spot of 132 μm in diameter in the case of the Green-Nanosecond laser, that gives a fluence of 1.90 J/cm^2^. In the case of the IR-Picosecond laser, the focal laser spot was 196 μm, which provides a fluence of 0.09 J/cm^2^. As a result of the laser ablation process, two different samples of Ag-NPs (G0 and IR0) in colloidal suspension were produced. Processing conditions are collected in Table 4.

All experiments were carried out by focusing the laser beam on the upper surface of the silver target. Likewise, during the laser ablation process, the laser beam kept moving relative to the target at a scanning speed of 50 mm/s. The LASL processing time of each sample was that required to obtain a concentration of 300 mg/L of silver nanoparticles.

Subsequently, part of each obtained colloidal solution was re-irradiated once and three times using the same laser source used before, leading to production of colloidal suspensions of resized Ag-NPs (G1, IR1 and G3, IR3 respectively). 

In order to conduct the bacteriological assays, the different groups of Ag-NPs (obtained according to Table 3) were immobilized on the surface of medical grade titanium (grade 2) discs with 25 mm of diameter and 1 mm thick (Sigma-Aldrich, Merck, MO, USA). These titanium discs were previously cleaned in ultrasonic bath with methanol for 15 min and rinsed gently with ultrapure deionized water in order to remove any contamination. The immobilization procedure of the Ag-NPs consisted of immersing three titanium discs in 50 mL of each colloidal solution obtained (see Table 3). Finally, the titanium discs completely covered by the colloidal solution were left to dry at room temperature. As a result of the evaporation, the nanoparticles remain immobilized onto the surface of the disc.

### 4.2. Sample Preparation and Characterization Techniques

The morphology and microstructure of the silver nanoparticles was evaluated by transmission electron microscopy (TEM) and high-resolution transmission electron microscopy (HRTEM) using a JEOL JEM 2010F (JEOL, Akishima, Japan) equipment. Additionally, selected area electron diffraction (SAED) was used for the evaluation of crystalline phases. For this purpose, drops of the different colloidal solutions were deposited on carbon-coated copper microgrids and on silicon substrates, and allowed to evaporate. Micrographs were obtained using 200kV accelerating voltage and a slow digital camera scan.

By comparing the interplanar distances measured in the SAED diffraction patterns with those obtained from the diffraction patterns of the ICDD database (JCPDS), the identification of the phases present in the nanoparticles was achieved.

The size distribution, together with the mean diameter and standard deviation of the silver nanoparticles, were obtained by using the Origin 8 software (OriginLab Co., Northampton, MA, USA) after measuring the diameter of about 400 nanoparticles. For this, different TEM micrographs of random areas of each surface were used.

The Z-Potential (ζp) measurements were obtained with a Zetasizer Nano ZS ZEN3600 (Malvern Panalytical, Malvern, UK). The cuvette used in the measuring process was a DTS1060 capillary cell gently cleaned with deionized water before each ζp measurement. Thereafter, the cell was filled with each sample completely and slowly in order to avoid the formation of air bubbles inside.

A Hewlett Packard HP 8452 spectrophotometer was used to obtain the UV-VIS absorption spectrum of the different colloidal solutions obtained. For this, duly clean quartz cuvettes were used, measuring the absorption spectrum in the range of 190 to 800 nm. 

Additionally, a field emission scanning microscope (FESEM, JEOL JSM 6700F) was used to obtain images of the immobilized nanoparticles on the surface of the titanium discs.

### 4.3. Bacterial Adhesion and Biofilm Formation Experiments

Titanium discs decorated with immobilized Ag-NPs corresponding to the six different NPs groups (see Table 3), were compared against controls of bare titanium samples. The antimicrobial properties were studied by the incubation of coupons with *Staphylococcus aureus* or mixed oral bacteria. In the last case, a highly reproducible in vitro multispecies biofilm model, recently developed, was used [79]. This model includes four relevant oral bacterial species involved in the development of peri-implantitis: *Streptococcus oralis*, *Actinomyces naeslundii*, *Veillonella dispar,* and *Porphyromonas gingivalis*. The bacterial adhesion and biofilm formation were studied as previously described [79,80]. All experiments were performed in triplicate.

The biofilm-forming collection strain *Staphylococcus aureus* ATCC 29213 was cultured overnight in brain heart infusion medium (BHI; CONDA, Spain) at +37 °C in 5% CO_2_ atmosphere. Bacteria were then suspended and diluted in BHI to the optical density (OD600) of 0.01 measured by an HELIOS beta Spectrophotometer, (Thermo Electron Corporation, Thermo Fisher Scientific, Waltham, MA, USA). 

The oral bacterial species *Streptococcus oralis* ATCC 9811, *Actinomyces naeslundii* DSM 43013, *Veillonella dispar* DSM 20735 and *Porphyromonas gingivalis* DSM 20709 were acquired from the American Type Culture Collection (ATCC) and the German Collection of Microorganisms and Cell Cultures (DSZM). Each oral bacteria were individually cultured overnight in BHI supplemented with 10 µg/mL vitamin K (Alfa Aesar, Thermo Fisher Scientific, Waltham, MA, USA) at 37 °C in anaerobic conditions for 48 h in a static model. Bacteria were then suspended and diluted in BHI to OD600 of 0.1. The bacterial dilutions were mixed equally and diluted in BHI with vitamin K to OD600 of 0.01 [79]. 

The titanium discs were placed into the bacterial suspension of *S. aureus* or the oral multispecies flora and incubated for 90 min to analyse planktonic bacterial adhesion or for 48 h to study biofilm formation in a static model, where, after 24 h of incubation, BHI medium was changed and incubated 24 h more.

Afterwards, the discs were rinsed three times with Dulbecco’s phosphate buffer saline (PBS) (Oxoid, Thermo Fisher Scientific, Waltham, MA, USA) to remove any non-adherent bacteria. Dried discs were stained using Live/Dead^®^ BacLight Bacterial Viability Kit (Invitrogen, Eugene, OR, USA). Stains were performed according to the instructions provided by the manufacturer. After staining, discs were analysed for bacterial adhesion using a fluorescence microscope Leica DM16000 B (Leica Microsystems, Wetzlar, Germany) or for biofilm formation using confocal laser scanning microscope (CLSM) (SP-5, Leica Microsystems, Wetzlar, Germany) [80].

#### 4.3.1. Bacterial Adhesion

On each disc, eight fields were viewed and photographed with Leica CFC420 digital camera (Leica Microsystems, Wetzlar, Germany) under a fluorescence microscope at ×20 magnification. The covered surface by *S. aureus* or multispecies oral bacteria was studied by analysing 24 microphotographs for each material using the ImageJ software (National Institute of Health, Bethesda, MD, USA).

#### 4.3.2. Biofilm Formation

On each disc, five fields were viewed and photographed with an automated confocal laser scanning microscope (CLSM) (SP-5, Leica Microsystems, Wetzlar, Germany) at ×20 magnification, as described elsewhere [81]. In brief, SYTO9 signals were detected using a multi-wavelength argon laser (excitation wavelength 488 nm and an emission range of 500–550 nm). An area of approximately 1.5 mm (X) × 1.5 mm (Y) was screened in 2 μm Z-intervals (Z-stack), and propidium iodine was measured with a 561 nm excitation wavelength and an emission range of 675–750 nm. The pinhole was adjusted to 2 μm. The 2D images of the green and red channel (24-bit) of each experiment were exported as two-dimensional bitmap images numbered (postfix) according to their Z-layer number with a size of 1024 × 1024 pixels and a resolution of 72 dpi. The thickness of the biofilm in these five predefined points/discs was associated with Z-value of this exported two-dimensional image. The surface area covered with adhered bacteria was calculated using the ImageJ software.

#### 4.3.3. Statistical Analysis

The analysis of the data obtained was carried out using the Statistical Package for the Social Sciences (SPSS) from IBM, version 22.0. This is a software program used by researchers of different fields for quantitative analysis of complex data. Data were reported as (mean ± SD). We used nonparametric statistical tests. When two samples were involved, the Mann–Whitney or the Wilcoxon tests were utilized, whereas the Kruskal–Wallis test was selected when the number of samples was higher than two. 

## 5. Conclusions

In this work, we demonstrated the effectiveness of silver nanoparticles produced by laser ablation and re-irradiation in liquid, against the formation of multispecies biofilms involved in the development of peri-implantitis of oral implants. This multispecies biofilm model includes the highly relevant oral bacterial species: *Streptococcus oralis*, *Actinomyces naeslundii*, *Veillonella dispar,* and *Porphyromonas gingivalis*. To compare the efficacy of Ag-NPs against biofilm formation, biofilms from a single bacterium (*Staphylococcus aureus*) and biofilms from multiple species were tested, showing outstanding antibiofilm activity in both cases. 

Since Ag-NPs with smaller sizes achieve a higher biofilm inhibition, triple laser ablation technique could be applied to synthesize other type of nanoparticles with enhanced antimicrobial properties. That could be an alternative to silver in view of the potential development of bacterial resistance to silver, due to the extensive use of this substance as an antimicrobial agent.

## Figures and Tables

**Table 1:** Z-Potential values.

Sample	G0	G1	G3	IR0	IR1	IR3
Z-Potential (mV) day 0	−24.5	−8.75	−2.33	−20.7	−18.9	−11.8

**Table 2:** The d-spacing as measured from selected area electron diffraction (SAED) performed on Ag nanoparticles obtained by (G0), (IR0) laser ablation, (G1), (IR1) by one re-irradiation, (G3), (IR3) by three re-irradiations, using the Green-nanosecond laser and the IR-picosecond one, respectively, and compared to those of metallic Ag.

Measured d-Spacing (nm)						
G0	G1	G3	IR0	IR1	IR3	Ag [hkl]
0.236	0.234	0.235	0.239	0.239	0.235	0.2359 (1 1 1)
0.209	0.208	0.214	0.192	0.222	0.204	0.2044 (2 0 0)
-	-	-	0.155	-	0.144	0.1445 (2 2 0)
-	-	-	-	-	0.123	0.123 (3 1 1)
-	0.116	-	-	-	0.118	0.11796 (2 2 2)

**Table 3:** Ag-NPs produced and analysed.

Sample	Laser Source	Treatment
G0	Green—nanosecond	As ablated
G1	Green—nanosecond	1 time re-irradiation
G3	Green—nanosecond	1 times re-irradiation
IR0	Infrared—picosecond	As ablated
IR1	Infrared—picosecond	1 time re-irradiation
IR3	Infrared—picosecond	1 times re-irradiation

**Table 4:** Laser parameters.

Laser Source	Wavelength (nm)	Pulse Duration (ns)	Pulse Frequency (kHz)	Pulse Energy (mJ)	Scanning Speed (mm/s)
Green—nanosecond	532	14	20	0.26	50
Infrared—picosecond	1064	0.8	200	0.03	50

## Data Availability

Not applicable.
